# Concentration dependent effect of calcium on brain mitochondrial bioenergetics and oxidative stress parameters

**DOI:** 10.3389/fnene.2013.00010

**Published:** 2013-12-18

**Authors:** Jignesh D. Pandya, Vidya N. Nukala, Patrick G. Sullivan

**Affiliations:** Department of Anatomy and Neurobiology, Spinal Cord and Brain Injury Research Center, University of KentuckyLexington, KY, USA

**Keywords:** brain mitochondria, calcium, mitochondrial bioenergetics, enzyme activities, membrane permeability transition (mPT), membrane potential (ΔΨ), reactive oxygen species (ROS), oxidative damage

## Abstract

Mitochondrial dysfunction following traumatic brain and spinal cord injury (TBI and SCI) plays a pivotal role in the development of secondary pathophysiology and subsequent neuronal cell death. Previously, we demonstrated a loss of mitochondrial bioenergetics in the first 24 h following TBI and SCI initiates a rapid and extensive necrotic event at the primary site of injury. Within the mitochondrial derived mechanisms, the cross talk and imbalance amongst the processes of excitotoxicity, Ca^2+^ cycling/overload, ATP synthesis, free radical production and oxidative damage ultimately lead to mitochondrial damage followed by neuronal cell death. Mitochondria are one of the important organelles that regulate intracellular calcium (Ca^2+^) homeostasis and are equipped with a tightly regulated Ca^2+^ transport system. However, owing to the lack of consensus and the link between downstream effects of calcium in published literature, we undertook a systematic *in vitro* study for measuring concentration dependent effects of calcium (100–1000 nmols/mg mitochondrial protein) on mitochondrial respiration, enzyme activities, reactive oxygen/nitrogen species (ROS/RNS) generation, membrane potential (ΔΨ) and oxidative damage markers in isolated brain mitochondria. We observed a dose- and time-dependent inhibition of mitochondrial respiration by calcium without influencing mitochondrial pyruvate dehydrogenase complex (PDHC) and NADH dehydrogenase (Complex I) enzyme activities. We observed dose-dependent decreased production of hydrogen peroxide and total ROS/RNS species generation by calcium and no significant changes in protein and lipid oxidative damage markers. These results may shed new light on the prevailing dogma of the direct effects of calcium on mitochondrial bioenergetics, free radical production and oxidative stress parameters that are primary regulatory mitochondrial mechanisms following neuronal injury.

## Introduction

Mitochondria act as a biological switch in determining a cell's fate. They provide the necessary energy for cell life and in cases of noxious stimuli cause cell death by necrosis and/or apoptosis. The physiological function of mitochondria is to produce adenosine triphosphate (ATP), the energy currency of the cell, through Krebs cycle and electron transport chain (ETC) activity. This ATP is utilized for various biological reactions inside the cell. Mitochondria are also involved in calcium (Ca^2+^) homeostasis. However, during ATP production, mitochondria also produce reactive oxygen/nitrogen species (ROS/RNS) as a by-product, mainly in the form of superoxide. Mitochondria are also equipped with anti-oxidant molecules and enzymes, and repair mechanisms. However, during pathophysiological conditions such as neurodegeneration and/or neurotrauma, the balance between ROS and anti-oxidants is tipped causing oxidative stress and Ca^2+^ dysregulation. This leads to mitochondrial dysfunction, triggering a signaling cascade resulting ultimately in cell death (Pedersen, 1999; Duchen, 2000; Kroemer and Reed, 2000; Newmeyer and Ferguson-Miller, 2003; Smaili et al., 2003; Sullivan et al., 2005; Wallace, 2005; Geddes and Sullivan, 2009).

The brain is a unique organ as it consists of a heterogeneous composition of cell types (neurons and glial cells) with distinct structural (cortex, hippocampus, striatum etc.) regions and functional (motor vs. sensory vs. memory) areas. It has low levels of stored glycogen; almost exclusively utilizes glucose as its energy source under normal conditions and consumes about 20% of total body oxygen even though it constitutes only 2% of total body mass. In addition, it contains high amounts of unsaturated fatty acids along with a high iron content, and has been found to be low in antioxidant defenses making it a prime target for oxidative stress (Floyd and Hensley, 2002).

Calcium homeostasis is critical for cell survival, human health and diseases (Berridge et al., 2000; Sullivan et al., 2005; Geddes and Sullivan, 2009; Nicholls, 2009; Pivovarova and Andrews, 2010). Levels of Ca^2+^ are maintained in the cytoplasm at a resting level of about 100 nM, and can rise up to 1 μM upon activation, as opposed to an extracellular concentration of approximately 1.2 mM (Berridge et al., 2000). In contrast, the mitochondrial concentration of Ca^2+^ responds to increases in the cytoplasmic concentrations such that the mitochondrial load is 10,000 fold higher due to the electrogenic, membrane potential driven uptake via the Ca^2+^ uniporter. Calcium exhibits wide-ranging spatial and temporal dynamics resulting in an extensive, but highly regulated, Ca^2+^ signaling network (Carafoli et al., 2001). At any given time, intracellular Ca^2+^ levels are maintained by a balance of “on” reactions enabling Ca^2+^ to enter the cell and “off” reactions resulting in its extrusion from the intracellular space, and the variations of on/off reactions generate Ca^2+^ transients. The “on” reactions are activated primarily by the flow of Ca^2+^, down its concentration gradient from the extracellular space, upon membrane depolarization via voltage-operated channels (VOC that include L-, P/Q-, N-, R-, and T-type channels) or agonist binding (glutamate, ATP, acetylcholine) via receptor-operated channels [ROC; ex: N-methyl-D-aspartate (NMDA) receptors] or store-operated channels (SOC) on the plasma membrane (PM). This initiates a signaling cascade engaging G-protein linked receptors, receptor tyrosine kinases (TyrK), and Phospholipase C (PLC). In a calcium-induced manner, Ca^2+^ can be released from internal stores such as endoplasmic reticulum (ER) predominantly via Ca^2+^-sensitive inositol 1,4,5-trisphosphate receptors (InsP_3_R) or ryanodine receptors (RyR) (Michelangeli et al., 2005).

Calcium is taken up by the mitochondria through a uniporter when the cytoplasmic Ca^2+^ level reaches around 1 μM and it is released when levels decrease below (Chalmers and Nicholls, 2003; Nicholls, 2005). Calcium is shown to regulate the citric acid cycle (TCA or Krebs cycle) by activating NAD^+^-dependent pyruvate dehydrogenase complex (PDHC), isocitrate dehydrogenase (ICDH) and α-ketoglutarate dehydrogenase complex (KGDHC) resulting in increases of NADH levels (Denton et al., 1988; Wan et al., 1989) as well as ATP production by activating the ATP synthase (Territo et al., 2000). The slow release of Ca^2+^ that occurs also regulates LTP and LTD by maintaining higher than normal levels in the cytoplasm. These reactions that occur within the mitochondria that relate to bioenergetics take place in the 0.1 to 1 μM concentration range with fast kinetics on the order of milli-seconds and may provide a physiological relevance. It is hypothesized that calcium inside the mitochondrial matrix forms a Ca^2+^-phosphate compound that is readily dissociable, which keeps free matrix Ca^2+^ at low levels. At still higher concentrations (>1 μM), calcium could inhibit respiration, cause mitochondria to undergo mPT and release pro-apoptotic proteins leading to cell death (Gunter et al., 2004; Nicholls and Chalmers, 2004; Jemmerson et al., 2005).

Several lines of evidence suggest that perturbations in intracellular Ca^2+^ homeostasis are involved in mitochondrial mediated cell death. Previously, our laboratory has demonstrated a loss of mitochondrial bioenergetics in the first 24 h following traumatic brain and spinal cord injury (TBI and SCI). Thus, mitochondria play a pivotal role in the development of secondary pathophysiology and subsequent neuronal cell death. However, the sequence of events leading from mitochondrial Ca^2+^ overload to ROS mediated damage in the mitochondria are still unclear. In light of these findings and gaps therein, we measured the direct effects of calcium concentrations on mitochondrial respiration, enzyme activity, free radical generation and oxidative damage markers in isolated brain mitochondria. The current data may shed light on the disparity of results in the literature concerning the effects of Ca^2+^ on mitochondrial function and ROS production. Further, it may be helpful in designing novel therapeutic strategies on the basis of current research findings on mitochondrial dysfunction parameters as they are common pathologies following traumatic brain, spinal cord injuries and other neurodegenerative conditions.

## Methods

### Reagents

Mannitol, sucrose, bovine serum albumin (BSA), ethylene glycol-bis(2-aminoethylether)-N,N,N′,N′-tetraacetic acid (EGTA), hydroxyethyl piperazine-1-ethanesulfonic acid potassium salt (HEPES), potassium phosphate monobasic anhydrous (KH_2_PO_4_), magnesium chloride (MgCl_2_), malate, pyruvate, adenosine 5′-diphosphate (ADP), succinate, calcium chloride (CaCl_2_), L-Arginine (L-Arg), potassium chloride (KCl), and horseradish peroxidase (HRP) were purchased from Sigma-Aldrich (St. Louis, MO, USA). Dihydro dichlorofluorescin diacetate (H_2_DCF-DA), tetramethylrhodamine ethyl ester (TMRE) were purchased from Molecular Probes (Eugene, OR). oligomycin A, rotenone and carbonyl cyanide 4-(trifluoromethoxy) phenylhydrazone (FCCP) were purchased from Biomol (Plymouth Meeting, PA). Bicinchoninic acid (BCA) protein assay kit was purchased from Pierce (Rockford, IL).

### Calcium concentrations

Stocks (5 mM CaCl_2_ solution) were prepared fresh daily in distilled water followed by serial dilution i.e., 100, 200, 500, and 1000, 20,000 nmol/mg in respiration buffer before experiment. The final calcium concentrations i.e., 100, 200, 500, and 1000, 20,000 nmol/mg mitochondrial protein were alternatively expressed as 10, 20, 50, and 100, 2000 μM respectively.

### Isolation and purification of brain mitochondria

All experimental protocols involving animals were approved by the University of Kentucky Animal Use and Care Committee. This protocol contains modifications of previously described procedures (Brown et al., 2004; Nukala et al., 2006). All procedures were performed on ice throughout the protocol. Male Sprague-Dawley rats (~250–300 g) were decapitated and the brains were rapidly removed. The cortices were dissected out and placed in an all-glass dounce homogenizer containing five times the volume of isolation buffer with 1 mM EGTA (215 mM mannitol, 75 mM sucrose, 0.1% BSA, 20 mM HEPES, 1 mM EGTA, and pH is adjusted to 7.2 with KOH). The tissue was homogenized and mitochondria were isolated by differential centrifugation. The homogenate was centrifuged twice at 1300 × g for 3 min in an eppendorf micro-centrifuge at 4°C. The resulting supernatant was transferred and topped off with isolation buffer with EGTA and centrifuged at 13,000 × g for 10 min. The supernatant was discarded and the pellet was resuspended in 500 μL of isolation buffer with EGTA. The trapped mitochondria were released from synaptosomes after synaptosomal membranes were disrupted using a nitrogen cell bomb incubated at 1200 psi for 10 min to obtain maximal yield of total (synaptic and non-synaptic) mitochondria. The mitochondrial sample was further purified by percoll density gradient centrifugation. Stocks of 40, 24, and 30% percoll were prepared fresh in isolation buffer with EGTA. 3.5 ml of 24% stock was carefully layered over 3.5 ml of 40% in 13 ml ultraclear tubes. Equal volumes of 30% percoll was added to the sample to get a 4 ml of 15% final concentration and loaded gently on top of the 24% layer. The sample was centrifuged in fixed angle type SE-12 rotor at 30,400 × g for 10 min at 4°C in RC 5C Plus (Sorvall) high-speed centrifuge. Fraction 3, containing total mitochondria, formed at the interphase of 40 and 24% layers and was carefully removed and placed in fresh tubes. The tubes were topped off with isolation buffer without EGTA (215 mM mannitol, 75 mM sucrose, 0.1% BSA, 20 mM HEPES and pH is adjusted to 7.2 with KOH) and centrifuged in fixed angle SE-12 rotor at 16,700 × g for 15 min at 4°C in RC 5C Plus (Sorvall) high-speed centrifuge. The supernatants were carefully removed and the tubes were topped off with isolation buffer without EGTA and centrifuged again at 13,000 × g for 10 min at 4°C. The supernatants were carefully removed and the loose pellets at the bottom were transferred to microcentrifuge tubes. The tubes were topped off with isolation buffer without EGTA and centrifuged at 13,000 × g for 10 min at 4°C. The supernatants were carefully removed and the pellets were resuspended in 500 μL of isolation buffer without EGTA. The tubes were topped off with isolation buffer without EGTA and centrifuged at 10,000 × g for 5 min at 4°C to yield a tighter pellet. The final mitochondrial pellet was resuspended in isolation buffer without EGTA to yield a concentration of ~10 mg/ml. The protein concentration was determined using the BCA protein assay kit measuring absorbance at 562 nm with a BioTek Synergy HT plate reader (Winooski, Vermont).

### Electron microscopy

After the last centrifugation, some of the mitochondrial pellets were fixed in 4% glutaraldehyde overnight at 4°C before being embedded for electron microscopy. Next, the pellets were washed overnight at 4°C in 0.1 M sodiumcacodylate buffer, followed by 1 h secondary fixation at room temperature in 1% osmium tetroxide. Then, the mitochondrial pellets were rinsed with distilled water and dehydrated for 10 min each in 70, 85, 95%, twice in 100% ethanol and twice in propylene oxide. The pellets were placed in a 1:1 mixture of propylene oxide and Epon/Araldite resin and infiltrated overnight on a rotator. Next, 100% Epon/Araldite resin was added and rotated for 1 h at room temperature. Finally fresh resin was prepared and degassed using a vacuum chamber. The mitochondrial pellets were added to the flat molds, filled with fresh resin, and baked overnight at 60°C. The 90 nm sections were cut using an RMC MT-7000 ultra-microtome mounted on 150 mesh copper grids and stained with uranyl acetate and lead citrate. The sections were examined using a Zeiss 902 electron microscope (Pandya et al., 2009).

### Western blotting

Western blots for COX and VDAC were carried out as described previously (Brown et al., 2004; Nukala et al., 2006). Briefly, the various samples were diluted to 1 μg/μl in isolation buffer without EGTA and used for western blots. Sample buffer was added to the samples based on relative protein concentrations and boiled for 10 min. Samples (5–20 μg each) were separated by SDS-PAGE 3–8% Tris-acetate gels (NuPage), along with molecular weight markers (Multi-Marker, Invitrogen). Following SDS-PAGE, polypeptides were transferred electrophoretically onto 0.2 μm nitrocellulose membranes. Membranes were incubated at room temperature for 1–2 h in 5% non-fat milk in 50 mM Tris-saline containing 0.05% Tween-20 at pH 7.5 (TTBS). The blots were incubated overnight in the primary antibody in TTBS at room temperature. The primary antibodies used in study included monoclonal cytochrome c oxidase subunit IV (COX IV) at 1:20,000 from Molecular Probes (Eugene, OR, USA) and polyclonal voltage-dependent anion channel (VDAC) at 1:10,000 from Affinity Bioreagents. After overnight incubation in primary antibody, the membranes were rinsed three times in TTBS and incubated in secondary antibody for 1–2 hin either HRP-conjugated goat anti-mouse IgG (1:6000) for COX-IV, or HRP-conjugated goat anti-rabbit IgG (1:6000) for VDAC. The blots were rinsed thoroughly in TTBS and were developed using Pierce SuperSignal West Pico chemi-luminescent substrate and analyzed using Kodak Image-Station.

### Respiration studies

The respiratory activity of isolated mitochondria was measured using a Clark-type oxygen electrode (Hansatech Instruments, Norfolk, England) (Brown et al., 2004; Nukala et al., 2006) in the presence of different concentrations of calcium (0, 100, 200, 500, 1000 nmol/mg mitochondrial protein). Approximately 120–160 μg/mL of isolated mitochondria were suspended in a sealed, constantly stirred, and thermostatically-controlled chamber at 37°C in respiration buffer (125 mM KCl, 2 mM MgCl_2_, 2.5 mM KH_2_PO_4_, 0.1% BSA, 20 mM HEPES at pH 7.2). The rate of oxygen consumption was calculated based on the slope of the response of isolated mitochondria to oxidative substrates in the order- 5 mM pyruvate and 2.5 mM malate; 150 μM ADP; 2 μM of oligomycin; 2 μM FCCP; 1 μ M rotenone and 10 mM succinate (Pandya et al., 2007, 2009, 2011; Patel et al., 2009).

### Mitochondrial enzyme activities

For measurement of PDHC and Complex I enzyme activities, mitochondrial protein samples (10 μg/100 μl) were co-incubated with increasing calcium concentrations (0–1000 nmols/mg mitochondrial protein) range. Additionally, a higher calcium dose (20000 nmol/mg mitochondrial protein) was also co-incubated to verify calcium effects at extremely high concentrations. Complex I enzyme activity was determined by measuring the decrease in NADH fluorescence at 460 nm in the presence and absence of rotenone, as previously described (Sriram et al., 1998; Sullivan et al., 2004; Gash et al., 2008). 10 μg of mitochondrial protein was added to 25 mM KPO_4_ pH 7.2, 5 mM MgCl_2_, 1 mM KCN, 1 mg/ml BSA, and 150 μM NADH. Either 1 μl of 1 mM rotenone or 1 μl of 25 mM KPO_4_ was added so that with and without rotenone samples were compared. The reaction was started by the addition of 50 μM of coenzyme Q1. Enzyme activity was calculated by subtracting the change in NADH emission in the absence of rotenone from the change in NADH emission in the presence of rotenone at 30°C using 96-well plate BioTek Synergy HT plate reader (BioTek, Winooski, VT). Similarly, PDHC activity was measured as previously described using a BioTek Synergy HT plate reader (Winooski, VT, USA) (Starkov et al., 2004). 10 μg of mitochondrial protein were added into the buffer containing 50 mM KCl, 10 mM HEPES pH 7.4, 0.3 mM thiamine pyrophosphate (TPP), 10 μM CaCl_2_, 0.2 mM MgCl_2_, 5 mM pyruvate, 1 μM rotenone, and 0.2 mM NAD^+^. The reaction was started by the addition of 0.14 mM CoASH and increased NAD^+^ fluorescence was recorded for 1 min intervals at 30°C. The PDHC activity was calculated and expressed as percentage change as compared to control group.

### Fluorescent spectrometry to measure mitochondrial total ROS/RNS generation and membrane potential (ΔΨ)

Mitochondrial ROS/RNS levels and membrane potential were measured using a Shimadzu spectrofluorophotometer (RF-5301) as described previously (Brown et al., 2006; Pandya et al., 2011). Mitochondrial protein (100 μg) was added to a thermostatically controlled (37°C), constantly stirred cuvette in a total volume of 2 ml of 125 mM KCl buffer, containing 5 mM pyruvate and 2.5 mM malate, 150 μM ADP, 2 μM of oligomycin, 1 mM L-Arg, 10 μM H_2_DCF-DA (Ex: 485 nm, Em: 530 nm), 150 nM TMRE (Ex: 550 nm, Em: 575 nm) and 25 U/ml HRP. Each run was performed following a baseline reading of the buffer. Mitochondria, calcium and the substrates/inhibitors were added as indicated. The slope of DCF fluorescence was quantified for the respective conditions including baseline and expressed in arbitrary units.

Alternately, for an additional confirmation of ROS generation, we measured mitochondrial H_2_O_2_ and total ROS/RNS production using 1 μM Amplex Red (Ex: 530 nm, Em: 590 nm) and 10 μM H_2_DCF-DA (Ex: 485 nm, Em: 530 nm) in the Biotek Synergy HT 96 well plate reader as previously described (Chen et al., 2003; Pandya et al., 2007, 2011; Visavadiya et al., 2013). Isolated mitochondria (10 μg) were added to 100 μl of 125 mM KCl respiration buffer with 5 mM pyruvate and 2.5 mM malate as oxidative substrates and incubated at 37°C. Fluorescence of the oxidized probe was measured every minute over a 10 min period (H_2_O_2_ production) and rate of total ROS/RNS production was calculated as the rate of Amplex Red fluorescence increase over 15–30 min incubation. Background control values (reagent and mitochondrial blanks) were subtracted from the results to get the slope for each experiment as calculated and expressed in arbitrary fluorescence units.

### Slot-blots for oxidative markers

Samples used for respiration were centrifuged at 10,000 g for 5 min. Buffer containing 0.1 M PBS and protease inhibitor cocktail (Roche) was added to samples, then diluted to 200 ng/ml with the protease inhibitor cocktail in 0.1 M PBS. For oxidative damage determination, the OxyBlot Protein Oxidation Detection Kit (Chemicon) was used according to manufacturer's specifications. Samples were loaded at 1 ug/well onto the slot blot apparatus and transferred onto 0.2 μm thick nitrocellulose membrane under vacuum. The membranes were blocked for 2 h, with agitation, in 1% Casein/PBS at room temperature. Primary antibodies were diluted in 0.1% casein/PBST and used as follows—rabbit-α-DNP (Chemicon) at 1:1000, mouse-α-3NT (Upstate) at 1:2000, rabbit-α-HNE (Calbiochem) at 1:10,000. Blots were incubated in primary antibody overnight, with agitation, at 4°C. Blots were rinsed with PBST and incubated Goat-α-Mouse (Jackson, for 3-NT) and Goat-α-Rabbit (Upstate; for HNE & DNP) for 2 h, with agitation, at room temperature. Blots were rinsed with PBST, dried, scanned, and quantified using a Bio-Rad scanner (Patel et al., 2009).

### Statistical analysis

All results are expressed as means ± s.e.m. For statistical evaluation, One-way analysis of variance (ANOVA) was used to test for differences involving multiple experimental groups or Student's *t*-test was employed for data analysis involving only two groups. When required, Fisher's *post-hoc* test was used for pair-wise comparisons among multiple groups. Significance was set at *p* < 0.05 for all analyses.

## Results

### Characterization of mitochondrial preparations

**Figure 1:**
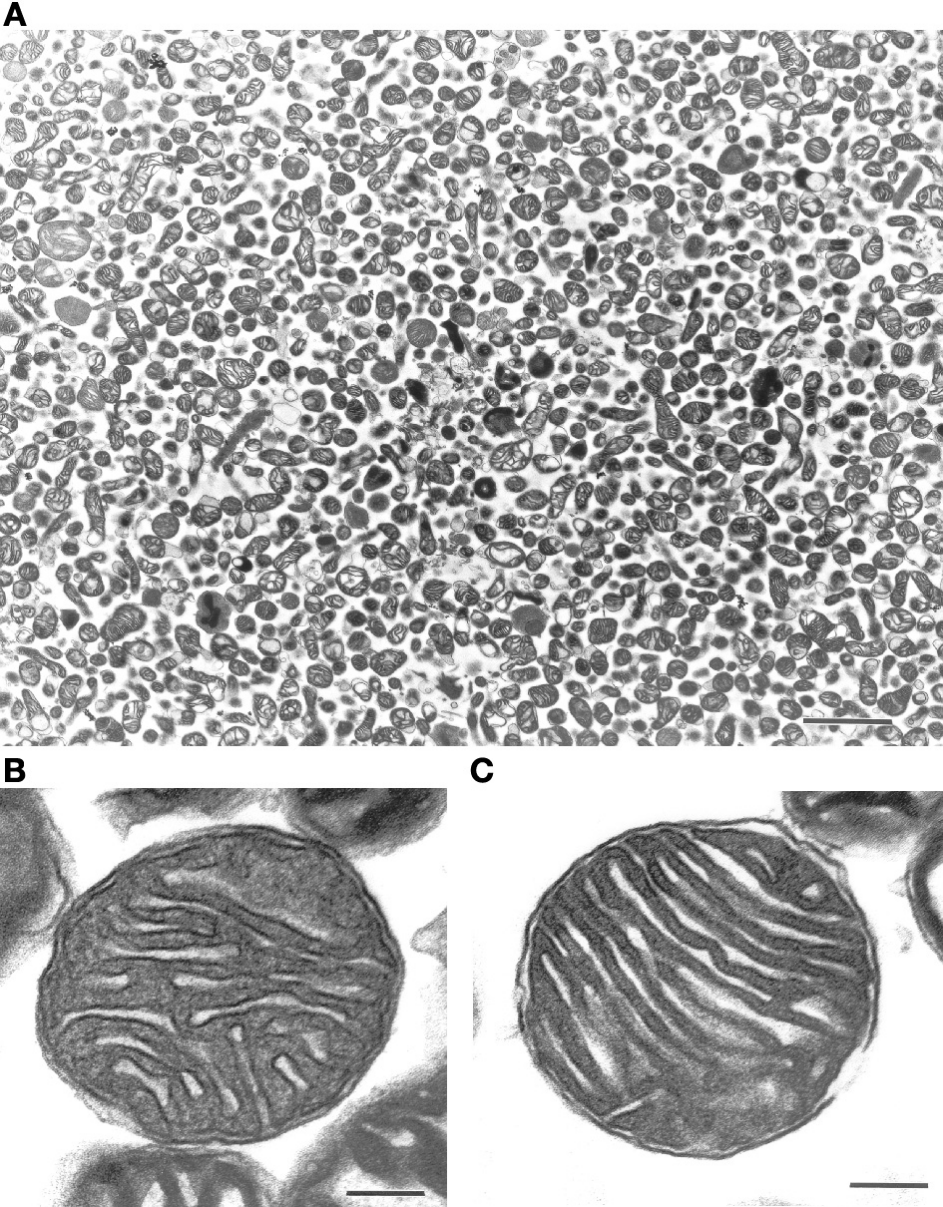
**Ultra-structure of mitochondria is maintained after percoll-purification**. Percoll-purified mitochondria were analyzed for purity and ultra-structure using electron microscopy (EM). The top panel **(A)** displayed freshly isolated mitochondrial photograph (Scale: 2 cm = 2 μm). The bottom panels **(B)** and **(C)** displayed intact outer and inner mitochondrial membranes along with maintained cristae (Scale: 1 cm = 0.1 μm).

**Figure 2:**
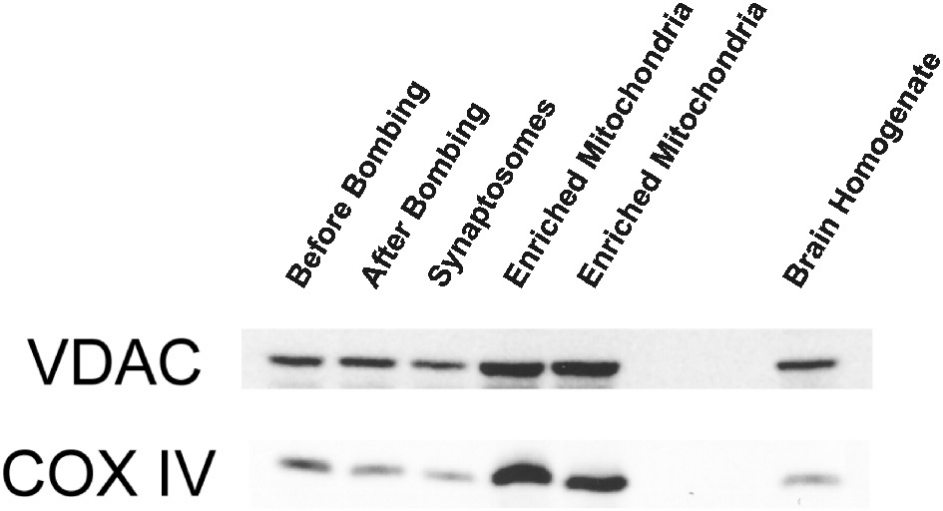
**Outer and inner mitochondrial membrane marker protein levels are enriched with percoll-purification**. Representative Western blots show mitochondrial markers in the various fractions collected during the isolation and purification procedure. The brain homogenate, synaptosomal, and crude mitochondrial pellets (before and after nitrogen bombing) fractions were collected for western blot analysis. The percoll-purified mitochondrial fractions demonstrate an enrichment of VDAC (outer membrane marker) and COX (inner membrane marker) protein levels compared to the brain homogenate and the crude mitochondrial fractions.

We initially characterized our mitochondrial isolation and purification methodologies by analyzing the ultra-structure of mitochondria and probing for mitochondrial markers. The electron-microscopy pictures indicate that the preparations predominantly contain mitochondria (Figure 1A). Mitochondria subjected to the percoll-purification method maintained their structures, including intact outer and inner mitochondrial membranes as well as tight cristae (Figures 1B,C). Mitochondrial marker proteins (VDAC and COX) were probed by western blots in the various fractions obtained during the sequential purification steps (Figure 2). We observed an enrichment of both outer (VDAC) and inner (COX) membrane markers in the percoll purified fraction whereas they are markedly lower in the sequential fractions collected during the preparation.

### Calcium and mitochondrial respiration

**Figure 3:**
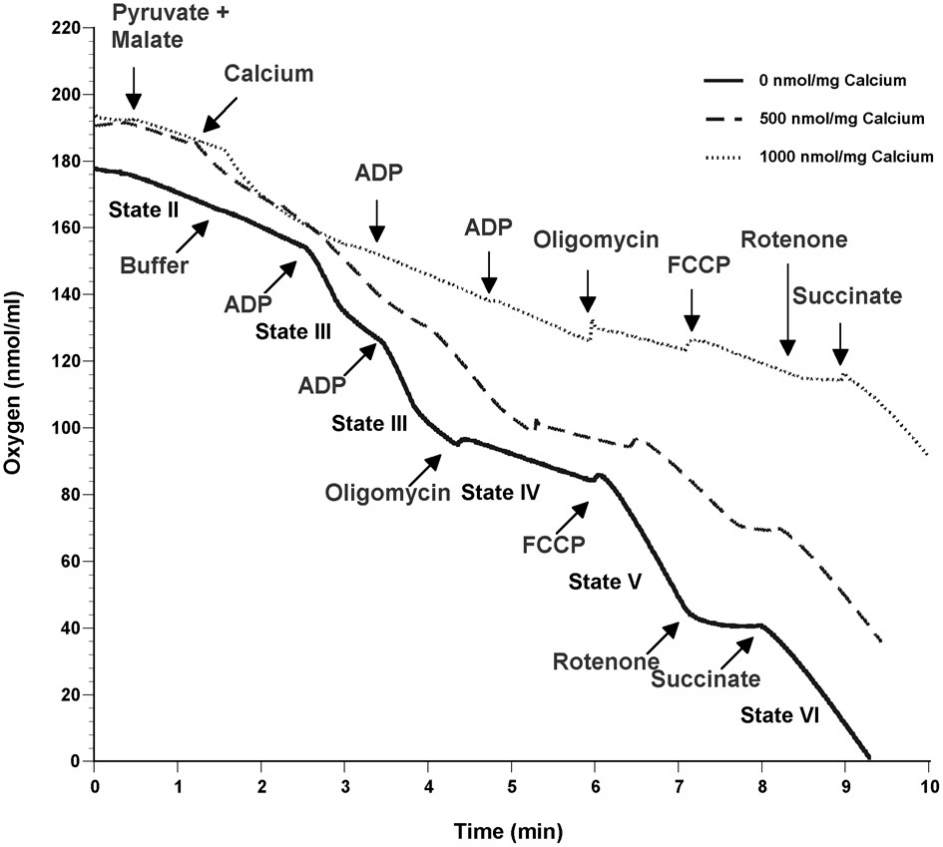
**Calcium inhibits mitochondrial respiration in a dose-dependent manner**. Representative traces show mitochondrial oxygen consumption with and without calcium. Mitochondrial respiration was carried out at 37°C by incubating mitochondria in respiration buffer and oxygen consumption of mitochondria was measured in response to addition of various substrates and inhibitors. Baseline respiration was termed as State I containing 125 mM KCl respiration buffer and mitochondria (25 μg). State II was initiated by addition of 5 mM pyruvate and 2.5 mM malate as substrates for Complex I of the ETC. Calcium concentrations (0–1000 nmol/mg mitochondrial protein) were added to the mitochondria at 1min followed by induction of State III respiration which was initiated by activation of ATP Synthase (Complex V) via the addition of ADP (150 μM). State IV occurs when ATP Synthase is inhibited by addition of oligomycin (2 μM). FCCP (2 μM) induces State V by uncoupling electron transport from oxidative phosphorylation. Rotenone (1 μM) was added inhibit complex I activity followed by succinate (10 mM) to measure respiration through Complex II. All respiration measurements were made in presence of 1mM L-Arg in KCl respiration buffer.

**Figure 4:**
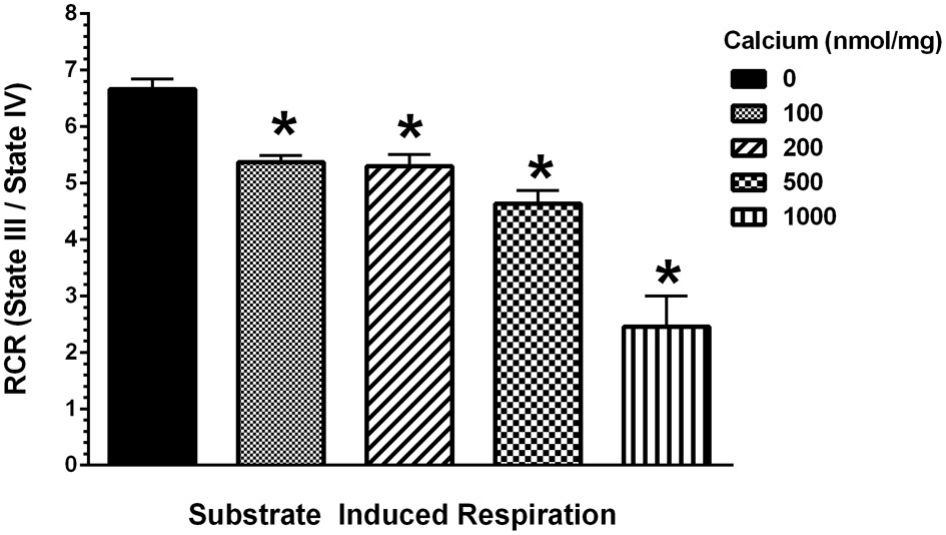
**Calcium decreases mitochondrial respiratory control ratio (RCR)**. Increasing concentrations of calcium were used at the doses (0–1000 nmol/mg mitochondrial protein) as indicated and the RCR were calculated as State III (presence of ADP) over State IV (absence of ADP and presence of oligomycin). The mitochondrial RCR in the presence of calcium was significantly lowered with increasing concentrations of calcium when compared to the RCR of mitochondria with no calcium addition. Data represent group means ± s.e.m.; *n* = 3 per group. One-Way ANOVA followed by Fisher's *post-hoc* test; ^*^p < 0.05 compared to 0 nmol/mg calcium.

**Figure 5:**
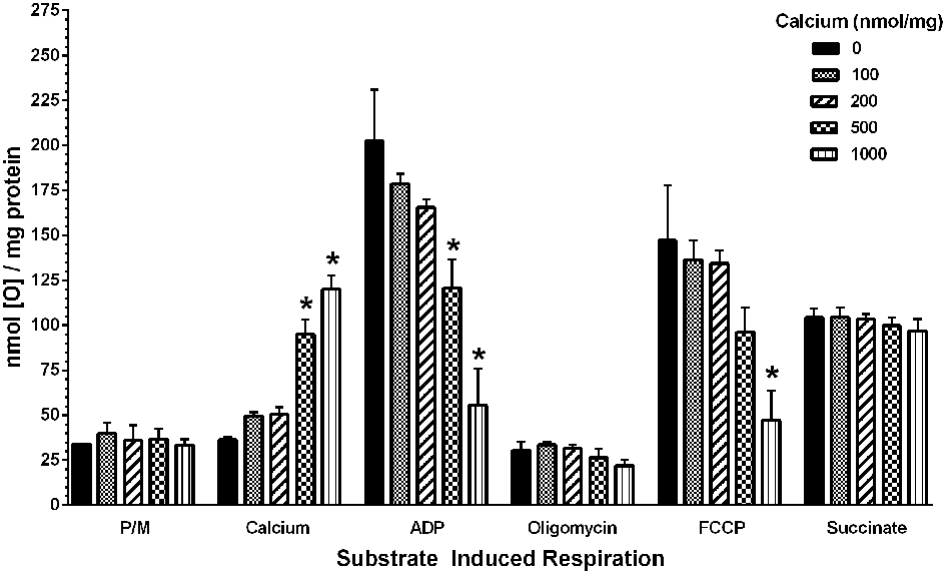
**Calcium decreases mitochondrial bioenergetics in a dose-dependent manner**. Increasing doses of calcium were added to the mitochondria after the addition of the complex I substrates pyruvate and malate. An increase in the nmol of oxygen consumed upon addition of calcium was observed, which reaches significance at 500 nmol/mg mitochondrial protein and later. Similarly, with increasing calcium concentrations, a trend in decreased bioenergetic response was observed in State III (ADP) and State V (FCCP) respiration whereas State IV(oligomycin) response remained unaltered. Calcium did not affect the complex II (Succinate) driven oxygen consumption. Data represent group means ± s.e.m.; *n* = 3 per group. One-Way ANOVA followed by Fisher's *post-hoc* test; ^*^p < 0.05 compared to 0 nmol/mg calcium.

**Figure 6:**
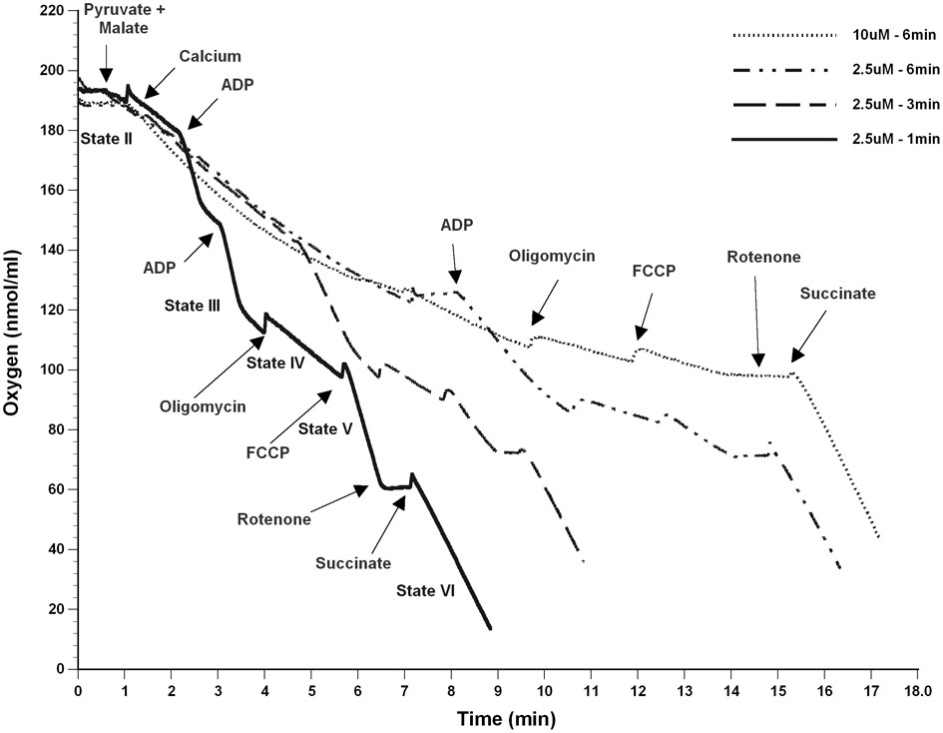
**Calcium decreases mitochondrial bioenergetics in a time-dependent manner**. Mitochondria (25 μg) were co-incubated with calcium after the addition of the complex I substrates (pyruvate and malate) for increasing amounts of time (1–6 min). An increase in the nmol of oxygen consumed upon addition of calcium was observed, while State III (ADP) driven respiration was inhibited with longer incubations with calcium (25 nmols/mg mitochondrial protein = 2.5 μM). When a higher concentration of calcium was added (100 nmols/mg mitochondrial protein = 10 μM), the inhibitory effect was more pronounced. However, as seen previously in Figure 5, calcium did not affect the complex II (Succinate) driven oxygen consumption. *n* = 2 per group.

**Figure 7:**
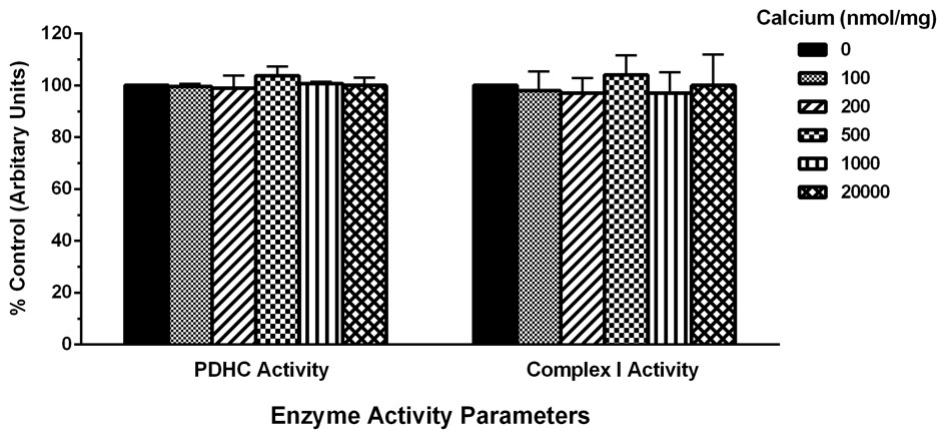
**Calcium does not alter mitochondrial enzyme activities**. For measurement of PDHC and Complex I enzyme activities, mitochondrial protein samples (10 μg/100 μl) were co-incubated with increasing calcium concentrations (0–20,000 nmols/mg protein) range. For both PDHC and Complex I assays, NAD^+^ or NADH (Ex: 360 nm, Em: 460 nm) dependent fluorescence change was recorded as an end-point measurement in multiple wells across time. Both PDHC and Complex I enzyme activities remained identical in calcium treated groups as compared to control group. Data are expressed as percent change in fluorescence ± s.e.m.; *n* = 3 per group. One-Way ANOVA followed by Fisher's *post-hoc* test.

To determine the possible effects of Ca^2+^ on mitochondrial function, we measured mitochondrial oxygen consumption using a Clark-type electrode and observed a Ca^2+^-mediated, significant reduction of mitochondrial respiration (Figure 3). The reduction in respiratory control ratio (RCR; State III/State IV) was dose-dependent (Figure 4). We then analyzed each of the substrate/inhibitor induced respiration states (Figure 5). We observed that Complex I (pyruvate/malate as substrates), NADH-linked respiration was affected specifically by calcium in a dose-dependent manner. We observed higher oxygen consumption upon addition of calcium accompanied by a reduction in State III respiration (in presence of ADP). However, State IV respiration (in presence of oligomycin- Complex V inhibitor) was not affected, indicating that the concentrations of Ca^2+^ utilized were below the levels to initiate mPT. There was also a trend of reduction in maximal oxygen consumption upon addition of the uncoupler FCCP. There were no changes observed in the presence of rotenone (Complex I inhibitor) or Complex II driven oxygen consumption (succinate as substrate). We observed an incubation time-dependent effect of calcium on the mitochondria in State III, State IV, and State V respiration. Interestingly, we again did not observe changes in succinate-driven oxygen consumption (Figure 6). Additionally, we measured mitochondrial Krebs cycle enzyme PDHC and ETC Complex I enzyme activities to measure direct effects of calcium on enzymes that contribute directly to the production and utilization of NADH metabolites in mitochondria. There were no changes observed in PDHC and Complex I enzyme activities in the presence of calcium concentrations that inhibited respiration in isolated mitochondria (Figure 7).

### Calcium and mitochondrial H_2_O_2_ production

**Figure 8:**
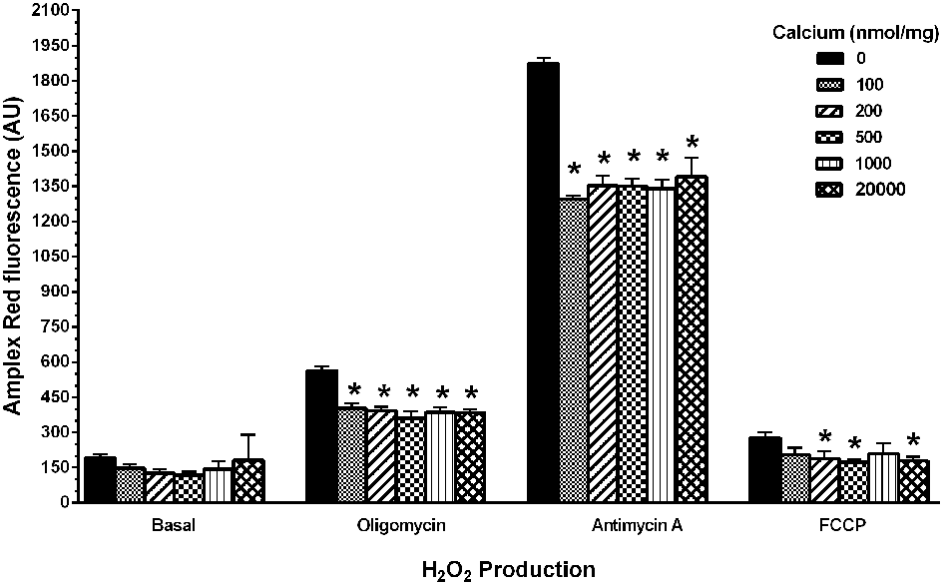
**Calcium decreases mitochondrial H_2_O_2_ production**. Mitochondrial hydrogen peroxide production was measured as various doses of calcium (0–20,000 nmols/mg mitochondrial protein) were co-incubated with energized mitochondria (10 μg/100 μl) in a 125 mM KCl respiration buffer (plus 5 mM pyruvate and 2.5 mM malate) for 0–10 min period at 30°C under four different assay conditions (Basal, oligomycin, antimycin A, FCCP). The 1 μM Amplex Red (Ex: 485 nm, Em: 530 nm) and 0.25 U HRP dependent fluorescence were recorded and differences in the fluorescence units for 10 min are expressed as arbitrary fluorescence units. The H_2_O_2_ production remained unchanged with calcium treatment in a basal respiratory condition where moderate mitochondrial membrane potential (ΔΨ) achieved. When H_2_O_2_ production with calcium concentrations was measured at a maximal membrane potential (ΔΨ) state (when treated with oligomycin and antimycin A); it significantly reduced with calcium treatment. Similarly, at minimal membrane potential (ΔΨ) state (when treated with FCCP); the H_2_O_2_ production remained significantly lower or unchanged. Data represent group means ± s.e.m.; *n* = 3 per group. One-Way ANOVA followed by Fisher's *post-hoc* test; ^*^p < 0.05 compared to 0 nmol/mg calcium.

**Figure 9:**
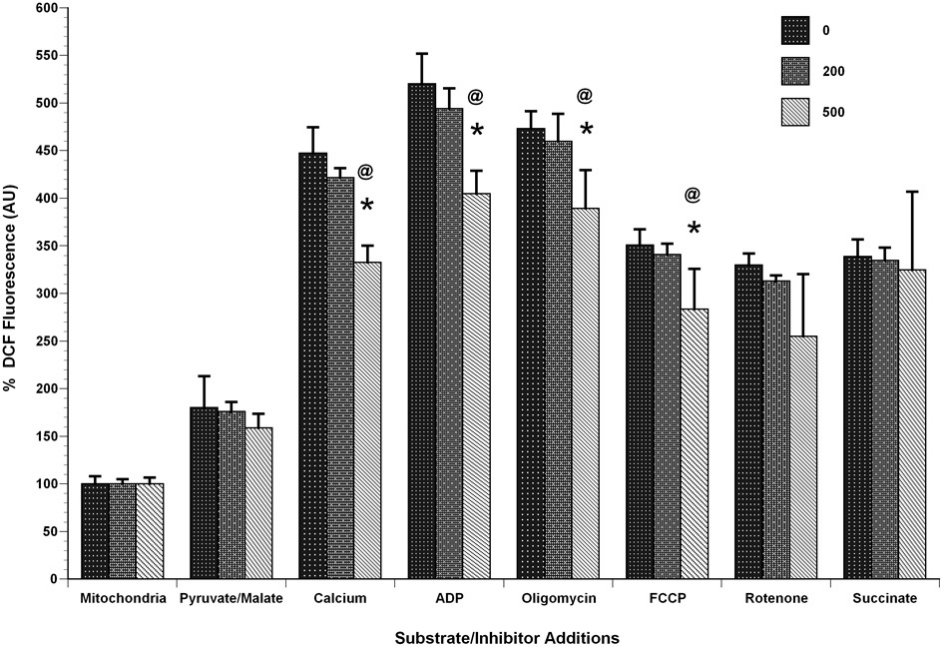
**Calcium decreases mitochondrial ROS/RNS levels**. Mitochondria (100 μg) were added to a constantly stirred cuvette in a total volume of 2 ml of 125 mM KCl buffer at 37°C containing 1 mM L-Arg, 10 μM H_2_DCF-DA (Ex: 485 nm, Em: 530 nm). Each run was performed with a baseline reading of buffer. Mitochondria, calcium and the substrates/inhibitors were added as indicated under identical mitochondrial bioenergetics conditions utilized in Figure 3. The slope of DCF fluorescence was quantified for the respective conditions including baseline and expressed as arbitrary units. A significant decrease in DCF fluorescence, as a measure of free radical generation, was observed in mitochondria treated with calcium (500 nmol/mg mitochondrial protein) compared control conditions. The decrease in DCF fluorescence was calcium dose-dependent and was observed throughout with additions of calcium, ADP, oligomycin, FCCP and rotenone but not with succinate addition. Data represent group means ± *SD*; *n* = 3 per group. One-Way ANOVA followed by Fisher's *post-hoc* test; ^*^p < 0.05 compared to 0nmol/mg calcium; @*p* < 0.05 compared to 200 nmol/mg calcium.

To determine if calcium could induce H_2_O_2_ production in the isolated mitochondria, we used the indicator Amplex Red to measure H_2_O_2_ production in four assay conditions [(1) Basal (in KCl respiration buffer, no substrates or inhibitors present), (2) under conditions of highest (oligomycin), and (3) lowest (FCCP) membrane potentials (ΔΨ) and also in presence of (4) Complex III inhibitor (antimycin A)]. We observed a significant decrease in the fluorescence of mitochondria in presence of calcium (100–20,000 nmol/mg mitochondrial protein) compared to those without calcium under conditions that induced H_2_O_2_ production (Figure 8). In addition to this, we measured total ROS/RNS measurements using DCF fluorescence as an indicator (Figure 9) under similar experimental conditions. Under these conditions, we again observed a significant decrease in the total ROS/RNS in presence of calcium (500 nmol/mg mitochondrial protein) as compared control group.

### Calcium and mitochondrial membrane potential (ΔΨ)

**Figure 10:**
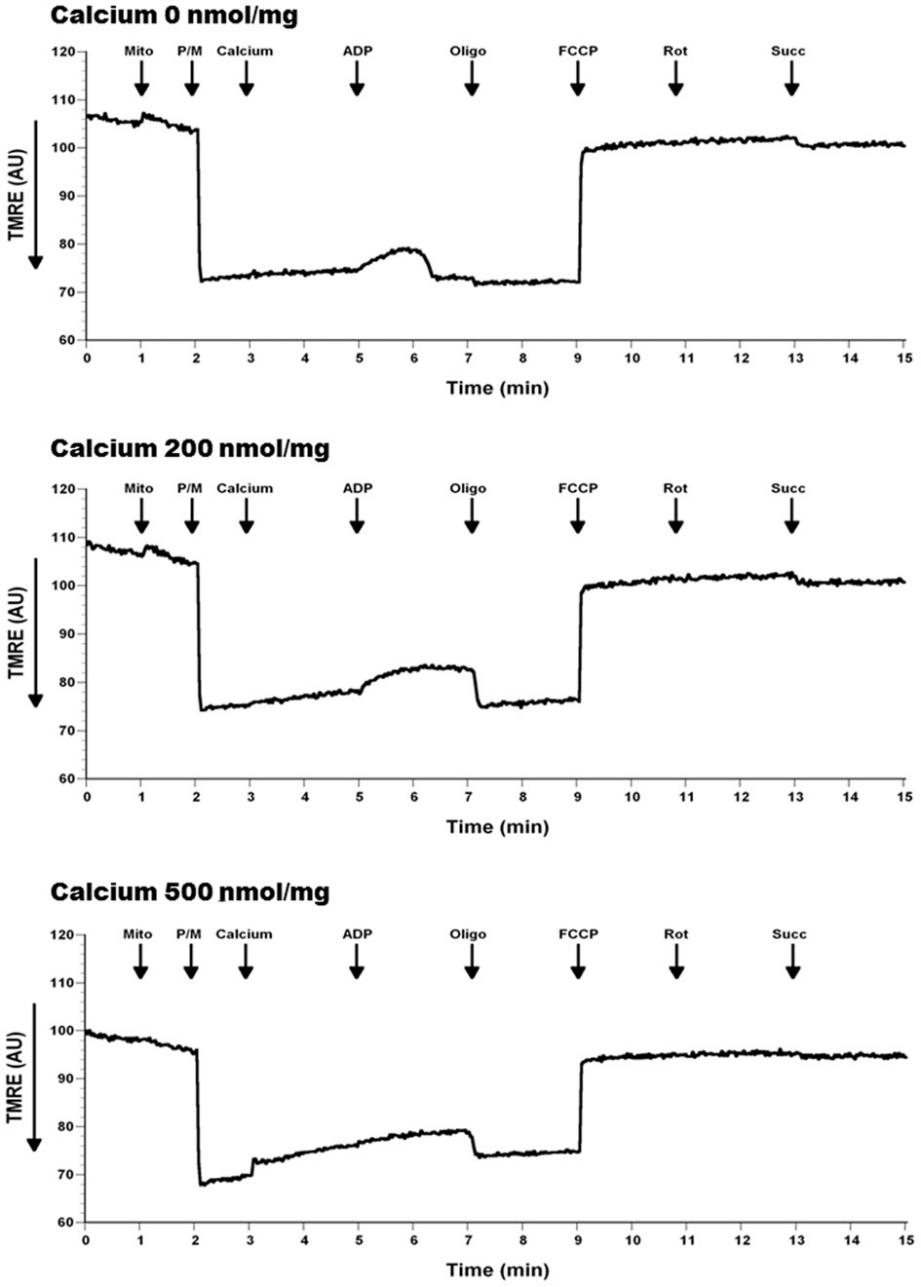
**Calcium decreases mitochondrial membrane potential (ΔΨ)**. Mitochondria (100 μg) were added to a constantly stirred cuvette in a total volume of 2 ml of 125 mM KCl buffer at 37°C containing 1 mM L-Arg, 150 nM TMRE (Ex: 550 nm, Em: 575 nm). Each run was performed with a baseline reading of buffer. Mitochondria, calcium and the substrates/inhibitors were added as indicated. An increase in TMRE fluorescence, as a measure of loss of mitochondrial membrane potential (ΔΨ), was observed when mitochondria treated with calcium compared to no calcium (slope of TMRE traces between 3 and 5 min). Additionally, this real-time loss of ΔΨ was calcium dose-dependent; and was sustained in the presence of ADP (slope of TMRE traces between 5 and 7 min). Additionally, a sharp bell shaped ADP response loss was evident with increasing calcium dose due to reflective prior loss of ΔΨ in presence of calcium. *n* = 3 per group.

To determine if calcium altered mitochondrial ΔΨ, we used the indicator TMRE, which is taken up by the mitochondria in a membrane potential dependent manner. As expected, due to the significant reductions in respiration, membrane potential was reduced by the addition of Ca^2+^ (Figure 10). Of interest are the observations that Ca^2+^ at 500 nmol/mg mitochondrial protein inhibits the mitochondrial depolarization that corresponds to ADP addition. Ca^2+^ at 200 nmol/mg mitochondrial protein also effects ADP phosphorylation evident by prolonged depolarization following ADP addition.

### Calcium and mitochondrial oxidative damage markers

**Figure 11:**
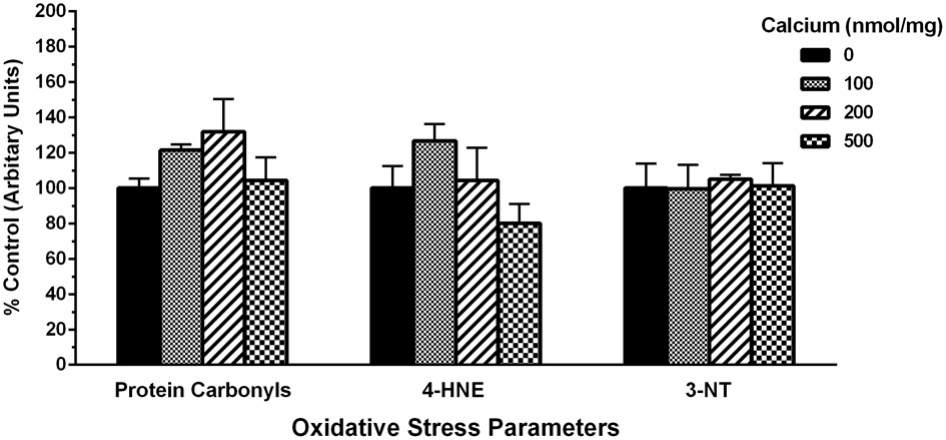
**Calcium does not alter protein and lipid oxidative stress parameters**. Using slot-blot assays, mitochondrial samples (200 ng/ml) were treated with increasing calcium concentrations and markers for protein oxidation (protein carbonyls), lipid peroxidation (4-HNE) and protein nitration (3-NT) and blot intensities assessed and expressed as percent increase compared to their levels in mitochondria without calcium. Protein carbonyls trended toward an increase at lower concentrations of calcium (100 and 200 nmol/mg mitochondrial protein) compared to higher concentrations (500 nmol/mg mitochondrial protein). However, these changes were not statistically significant compared to control (no calcium). No changes in the levels of 4-HNE or 3-nitrotyrosine (3-NT) were observed with any of the calcium concentrations used. Data represent group means ± s.e.m.; *n* = 3 per group; One-Way ANOVA.

Lastly, to assess the total extent of mitochondrial oxidative damage following Ca^2+^ exposure, we utilized slot-blots to measure changes in markers of protein oxidation, lipid peroxidation and protein nitration. We observed a trend for increases in the levels of mitochondrial protein carbonyls. However, no differences were observed with 4-hydroxynonenal (4-HNE) and 3-nitrotyrosine (3-NT) oxidative damage markers when mitochondria were co-incubated at the various concentrations of calcium (Figure 11).

## Discussion

The current study demonstrates that increasing calcium concentrations directly inhibit mitochondrial respiration in a dose and time-dependent manner. Additionally, mitochondrial free radical production was reduced at the levels of Ca^2+^ used in the present study. Moreover, while we did observe changes in mitochondrial levels of protein carbonyls (indicator of protein oxidation); we did not observe any significant increase in the levels of 3-Nitrotyrosine (3-NT; indicator of protein nitration) and 4-Hydroxynonenal (4-HNE; indicator of lipid peroxidation) oxidative damage markers in the presence of calcium. These observations in isolated brain mitochondria indicate that mitochondrial respiration failure is an immediate primary event following calcium exposure whereas free radical production and oxidative stress may become operative as secondary events after energy failure. These results suggest a possible temporal “cause vs. effect” relationship amongst mitochondrial mechanisms after calcium exposure.

Even in the present study, under *in vitro* or *ex vivo* conditions, it is apparent that mitochondrial bioenergetics and calcium homeostasis operate in a very complex interaction. *In vivo* these interactions are no doubt even more complex and involved other cellular organelles including the endoplasmic reticulum (ER) and cytosolic compartments. Therefore, there are multiple regulatory components that can also influence calcium homeostasis and mitochondrial respiration (Gnaiger, 2001; Gellerich et al., 2012, 2013; Llorente-Folch et al., 2013). It is also important to note the findings of Gellerich and colleagues who have demonstrated eloquently the effects of low levels (<6 nmols Ca^2+^/mitochondrial protein) Ca^2+^ on respiration in isolated brain mitochondria which increases respiration (Gnaiger, 2001; Gellerich et al., 2012, 2013).

In the current study, we isolated ultrapurified cortical mitochondria (synaptic and nonsynaptic) using percoll discontinuous gradient centrifugation. This means that the mitochondria isolated for our study are from a heterogeneous sources of cell types, chiefly from neurons and glial cells. Additionally, mitochondria isolated from the brain are most often contaminated by synaptosomes, formed by the pinched off pre- and post-synaptic ends of neurons, that can alter and complicate the interpretation of results obtained from such mitochondrial preparations (Anderson and Sims, 2000; Brown et al., 2006). Therefore, we employed a method to release the synaptic mitochondria using nitrogen disruption which increases the total yield of mitochondria (Brown et al., 2004), while avoiding the adverse effects of digitonin (Brustovetsky et al., 2002). We checked the purity of these mitochondrial preparations by electron microscopy and found them to have mostly mitochondria with preserved structures including intact cristae that lodge the ETC necessary for oxidative phosphorylation (Figure 1). Moreover, mitochondrial marker proteins such as VDAC for outer and COX for inner mitochondrial membranes are enriched in the mitochondrial fraction with the sequential purification steps (Figure 2) while minimizing contamination. Furthermore, we used only fresh mitochondria for the studies described in this paper, as frozen mitochondria exhibit decreases in mitochondrial bioenergetics (Nukala et al., 2006).

In our study, we added doses of Ca^2+^ (100–1000 nmol/mg mitochondrial protein) in respiration buffer and examined its and examined its effects on Complex I driven mitochondrial respiration. We observed that calcium induced mitochondrial dysfunction and the severity was dose-dependent (Figure 3), similar to previous reports (Brustovetsky et al., 2003; Zhou et al., 2005). Of interest is the fact that mitochondria subjected to density gradient centrifugation are more sensitive to Ca^2+^ effects than unpurified CNS mitochondria (Brustovetsky and Dubinsky, 2000). We observed significant decreases in RCR (Figure 4), as well as decreases in individual states, most noticeably in State III (coupled respiration in presence of ADP) and to a lesser extent in State V (maximal respiration in presence of FCCP) as seen in Figure 5.

Though the oxygen consumption increased initially upon addition of calcium and returned to State II respiration rates (Figure 5), the later response to ADP was markedly affected, and this could be due to the transient depolarizing effect experienced with calcium on membrane potential. In one study, mitochondria exposed to a low Ca^2+^ concentration (4 μM) resulted in a VDAC-mediated reversible cytochrome c release, whereas at a higher Ca^2+^ concentration (100 μM), there was mitochondrial inhibition due to mPT induced irreversible cytochrome c release (Schild et al., 2001). However, in the current study, no changes could be observed in Complex II (succinate as the substrate) driven oxygen consumption indicating that the calcium may target NADH dependent inhibition of mitochondrial respiration through inhibition of Complex I or upstream enzymes that produce NADH in the Krebs cycle. Dubinsky's group, upon addition of calcium, has shown that cytochrome c released was accompanied by mPT and rupture of outer mitochondrial membrane. However, the decline in mitochondrial respiration was attributed to inhibition of Complex I rather than loss of cytochrome c (Brustovetsky et al., 2002). Further, the inhibition of mitochondrial respiration was exacerbated with longer exposures to calcium (Figure 6), consistent with a previous study (Territo et al., 2000). In the present study, no indication of mPT activation is apparent given the lack of changes observed in state IV or complex II-driven respiration. This suggests that deleterious effects of calcium are induced immediately and sustained over a prolonged period of time that may activate other secondary regulatory pathways after calcium exposure.

To further probe the mechanism for this Complex I driven impairment in respiration, we directly measured enzyme activities of two NADH dependent enzymes, the Krebs cycle gate keeper enzyme PDHC and ETC gate keeper Complex I in presence of increasing calcium concentrations. We expected to see decreased enzyme activities since they regulate NADH production and utilization during mitochondrial respiration. Conversely, our data in Figure 7 indicates that both the PDHC and Complex I activities in presence of increasing calcium concentrations were not altered significantly and remained identical to control group values. This could be due to the fact that calcium may not inhibit those enzyme complexes directly. Alternatively, decreased activities of brain mitochondrial PDHC and Complex I reported by others in *in vivo* experiments in various neurological conditions may be due to oxidation of these enzymes (Sorbi et al., 1983; Schapira et al., 1989; Keeney et al., 2006; Gash et al., 2008; Andreazza et al., 2010). Furthermore, the doses used in these studies may not reflect the actual concentrations that these enzymes are exposed to in respiring mitochondria given the electrogenic nature of mitochondrial Ca^2+^ cycling.

Mitochondria are major sources of free radicals inside the cell due to leakage of electrons from ETC (Turrens, 2003), and calcium is shown to influence ROS/RNS generation in mitochondria by processes that are not completely understood (Camello-Almaraz et al., 2006). This is evident by the divergent reports on calcium increasing (Brustovetsky et al., 2003; Sousa et al., 2003; Hansson et al., 2008), decreasing (Starkov et al., 2002; Starkov and Fiskum, 2003; Gyulkhandanyan and Pennefather, 2004), or causing no changes (Votyakova and Reynolds, 2001, 2005; Tretter and Adam-Vizi, 2007) in the ROS/RNS levels of brain mitochondria. In general, processes of free radical formation, with respect to mitochondrial membrane potential (ΔΨ) dependence, are not well defined or understood. Conclusively, succinate usually in presence of ETC inhibitors tends to produce higher levels of ROS/RNS with calcium compared to pyruvate/malate/glutamate as substrates. Hence, it was of interest in the present study to assess calcium induced H_2_O_2_ and total ROS/RNS generation in presence of respiratory substrates and inhibitors that regulate mitochondrial ΔΨ. We used pyruvate and malate substrates for measurement of complex I driven and NADH dependent free radical productions when incubated in different calcium concentrations with isolated mitochondria. Also, we have included known ΔΨ regulators such as oligomycin, antimycin A and FCCP to understand the influence of calcium on ΔΨ dependent H_2_O_2_ production.

Our current data demonstrated a significant decrease in ΔΨ-dependent H_2_O_2_ production in the presence of calcium (in presence of substrates pyruvate-malate with either oligomycin, antimycin A, and FCCP). Additionally, no significant differences were observed in H_2_O_2_ level in basal respiratory conditions when no inhibitor and substrate were added (Figure 8). In another experimental protocol, we observed significant decreases in total ROS/RNS levels with calcium (500 nmol/mg mitochondrial protein) using an alternative free radical indicator, DCF (Figure 9). This was accompanied by a partial loss of membrane potential (Figure 10) as would be expected by the consumption of ΔΨ for mitochondrial uptake of calcium and subsequent inhibition of mitochondrial respiration, supporting our data and previous reports (Vergun and Reynolds, 2005).

Our lab has previously shown that non-synaptic mitochondria are able to buffer more calcium than synaptic mitochondria before losing the ΔΨ and undergoing swelling; but they do not exhibit differences in ROS/RNS levels (Brown et al., 2006; Naga et al., 2007). Those non-synaptic and synaptic mitochondria were able to sequester calcium concentrations i.e., 2500 nmols/mg mitochondrial protein (250 μM) and 1000 nmols/mg mitochondrial protein (100 μM) respectively, which is higher than the range used in the current set experiments (Figures 8, 9). One important difference here is the use of total mitochondria and the absence of ADP before the calcium challenge. It is known that the presence of ADP reduces mitochondrial membrane potential (ΔΨ) and therefore the amount of calcium sequestered before mPT formation (Andreyev et al., 1998). Another similar report has also demonstrated that until mPT occurs ROS is not released (Hansson et al., 2008), which was the reason we increased our calcium concentration range up to 20000 nmol/mg mitochondrial protein for this set of experiment (Figure 8). Under ΔΨ influenced experimental conditions, the H_2_O_2_ levels remained significantly lower as compared to control groups when treated across the entire range of calcium concentrations. In this experimental design, we added calcium concentrations (10–2000 μM) that cover the entire effective range that may or may not directly induce mPT (Figure 8). The results from our experiments indicate that inhibition of mitochondrial respiration via Ca^2+^ uptake inhibits mitochondrial ROS production by limiting the influx of substrates into the ETC which limits ΔΨ-dependent ROS production. Additionally, when assaying these experiments, it should also be kept in mind that Amplex Red and dichlorofluorescein (DCF) which measures a range of total ROS/RNS including H_2_O_2_ and ONOO^−^ are very susceptible to photo auto-oxidation and proper controls must be used for samples including darkened conditions, which we employed to obtain, are necessary to obtain reliable results (Possel et al., 1997; Ischiropoulos et al., 1999; Radi et al., 2001; Thomas et al., 2002).

Next we tested if the changes in H_2_O_2_ and total ROS/RNS levels observed with calcium translated into changes in markers of oxidative damage in the mitochondria (Figure 11). We measured the levels of protein carbonyls (an indicator of protein oxidation), 4-hydroxynonenal (4-HNE; indicator of lipid peroxidation). We observed differences in protein carbonyls and 4-HNE at lower concentrations of calcium indicating a trend toward an increase in oxidative damage but significance was not achieved in the present studies. Moreover, a wide variety of nitrated proteins have been found in the mitochondria indicating involvement of peroxynitrite (Riobo et al., 2001; Elfering et al., 2004) and therefore we measured the levels of 3-nitrotyrosine (3-NT; indicator for protein nitration). However, we did not observe any changes in levels of 3-NT at any of the calcium doses tested even in the presence of L- Arg (Figure 11). An absence of measurable or significant changes in oxidative damage markers does not necessarily mean a lack of H_2_O_2_ and total ROS/RNS involvement since we have used isolated rat cortical mitochondria for *in vitro* experiments in the presence of calcium. The methodology used measures the extent of total oxidative damage and not the individual oxidatively modified proteins (Kohen and Nyska, 2002; Tarpey et al., 2004). The short exposure time (less than 10 min) of mitochondria to calcium might be insufficient to elicit detectable changes in total ROS/RNS levels and/or oxidative damage markers. In fact, oxidative damage to certain key mitochondrial proteins, including those involved in Krebs cycle or ETC has been observed. While Complexes I and II of the ETC, creatine kinase are oxidatively inactivated by peroxynitrite, ATP synthase, aconitase, VDAC, and Mn-SOD are inactivated by nitration of tyrosine residues through the peroxynitrite decomposition products (Radi et al., 2002). The inactivation or inhibition of these specific proteins might be sufficient to inhibit mitochondrial respiration (ex: ATP Synthase) or amplify free radical generation (ex: Mn-SOD) and thereby contribute to mitochondrial dysfunction ultimately resulting in cell death.

Calcium induced mitochondrial dysfunction may be only one of the many mechanisms responsible for the global cellular oxidative stress and may require activation of the mPT (Hansson et al., 2008). Another possible mechanism is the cytosolic calcium induced activation of phospholipase A_2_ (PLA_2_) that mediates the rapid release of arachidonic acid (AA) and lysophospholipids. Some of the AA is converted to pro-inflammatory mediators such as prostaglandins, leukotrienes, and thromboxanes, collectively known as eicosanoids. AA cascade can lead to generation of free radicals resulting in lipid peroxidation thereby altering plasma membrane as well as mitochondrial membrane composition, fluidity and permeability. The mitochondrial proteins can be oxidized from such cytosolic sources of free radicals and nitrated by cytosolic NOS mediated NO^•^ and ONOO^−^ generation. Further, calcium activated cytosolic calpains can also degrade mitochondrial proteins thereby causing mitochondrial dysfunction. In the current study, involvement of cytosolic mechanisms that may influence mitochondrial structure and function were not tested.

In summary, our experimental analysis of dose and time dependent *in vitro* effects of calcium concentrations on mitochondrial bioenergetics and oxidative mechanisms may provide valuable clarifications in isolated mitochondria. Further it may be beneficial to design therapeutic strategies on the basis of mitochondrial dysfunction parameters as they are prominent in common pathologies following traumatic brain, spinal cord injuries and other neurodegenerative conditions.

## Author contributions

Drs. Pandya and Nukala designed and conducted experiments, performed analysis and prepared manuscript. Dr. Sullivan contributed and guided throughout the experimental planning and manuscript preparation.

### Conflict of interest statement

The authors declare that the research was conducted in the absence of any commercial or financial relationships that could be construed as a potential conflict of interest.
